# Assessing translational applicability of perineuronal net dysfunction in Alzheimer’s disease across species

**DOI:** 10.3389/fnins.2024.1396101

**Published:** 2024-04-30

**Authors:** Aarun S. Hendrickson, Kendra L. Francis, Asmit Kumar, Jaden P. Le, Jarrad M. Scarlett, C. Dirk Keene, David A. Tovar, Kimberly M. Alonge

**Affiliations:** ^1^Medicinal Chemistry, University of Washington, Seattle, WA, United States; ^2^Department of Medicine, University of Washington Medicine Diabetes Institute, Seattle, WA, United States; ^3^Department of Pediatric Gastroenterology and Hepatology, Seattle Children’s Hospital, Seattle, WA, United States; ^4^Department of Laboratory Medicine and Pathology, University of Washington, Seattle, WA, United States; ^5^Department of Psychology, Vanderbilt University, Nashville, TN, United States

**Keywords:** Alzheimer’s disease, chondroitin sulfate, perineuronal nets, translation, glycosmainoglycans, mass spectrometry, proteoglycans

## Abstract

In the context of aging and age-associated neurodegenerative disorders, the brain’s extracellular matrix (ECM) serves as a critical regulator for neuronal health and cognitive function. Within the extracellular space, proteoglycans and their glycosaminoglycan attachments play essential roles in forming, stabilizing, and protecting neural circuits throughout neurodevelopment and adulthood. Recent studies in rodents reveal that chondroitin sulfate-glycosaminoglycan (CS-GAG) containing perineuronal nets (PNNs) exhibit both structural and compositional differences throughout the brain. While animal studies are illuminating, additional research is required to translate these interregional PNN/CS-GAG variations to human brain tissue. In this perspective article, we first investigate the translational potential for interregional CS-GAG variances across species as novel targets for region-specific therapeutic development. We specifically focus on the observation that alterations in brain PNN-associated CS-GAGs have been linked with the progression of Alzheimer’s disease (AD) neuropathology in humans, but these changes have not been fully recapitulated in rodent models of this disease. A second highlight of this perspective article investigates whether AD-associated shifts in CS-GAGs in humans may be dependent on region-specific baseline differences in CS-GAG sulfation patterning. The current findings begin to disentangle the intricate relationships between the interregional differences in brain PNN/CS-GAG matrices across species, while emphasizing the need to better understand the close relationship between dementia and changes in brain CS-GAG sulfation patterns in patients with AD and related dementias.

## 1 Introduction

**Figure 1:**
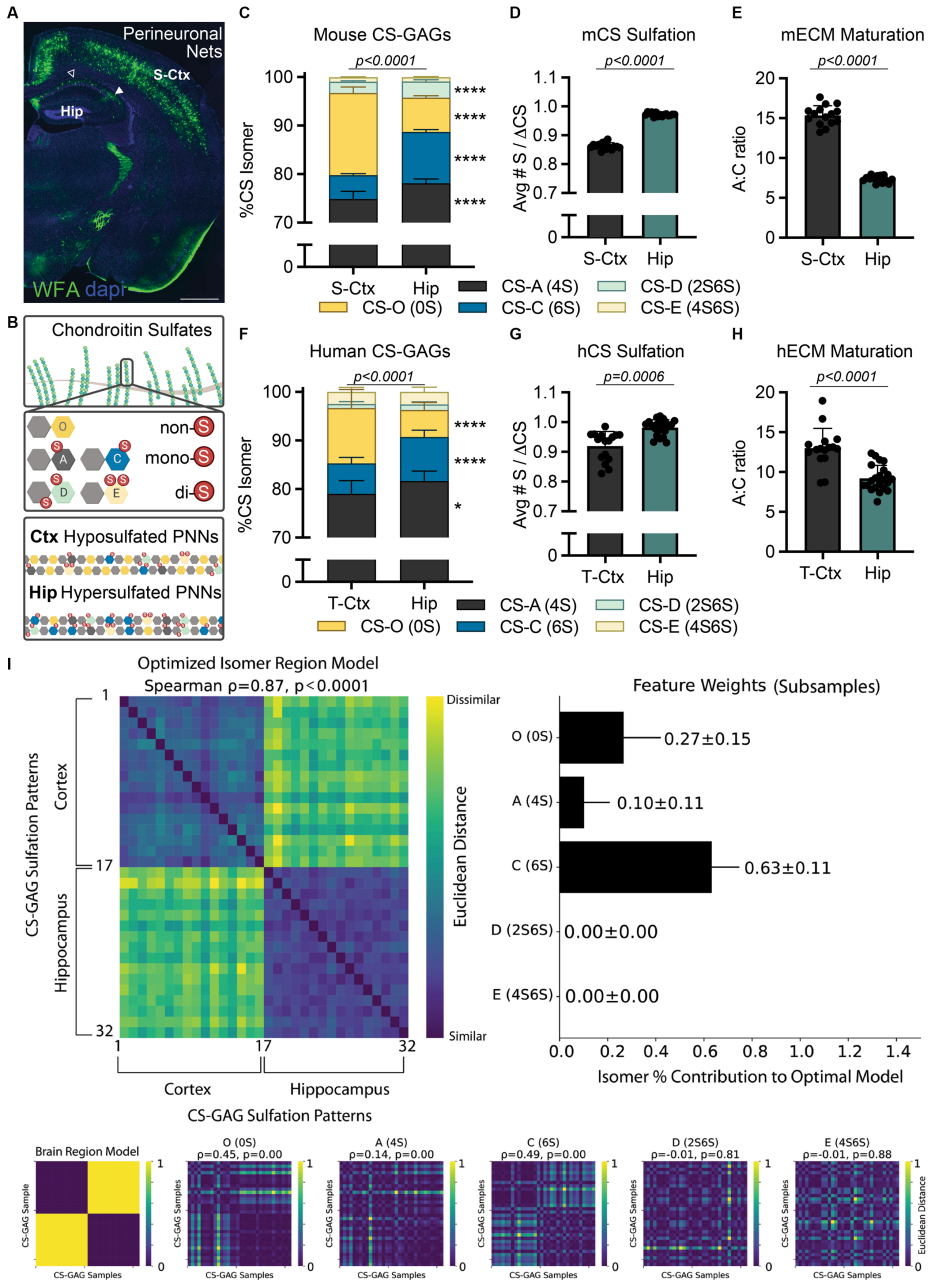
Translating interregional PNN/CS-GAG differences across species. **(A)** PNN CS-GAG labeling by WFA in a male, P42 mouse brain shows increased WFA+ PNNs in the somatosensory cortex compared to the adjacent dorsal hippocampus. Open arrow represents hippocampal CA1 and closed arrow FIGURE 1 (Continued)represented hippocampal CA2 subregions. Scale: 1 mm. **(B)** CS-GAGs are comprised of CS-O (0S), CS-A (4S), CS-C (6S), CS-D (2S6S), and CS-E (4S6S) isomers, which influence the overall charge of the extracellular environment based on sulfation (non-(0S), mono-(1S), and di-(2S) sulfated CS variants). LC-MS/MS of brain CS-GAGs in **(C–E)** mice (see (Scarlett et al., 2022)) and **(F–H)** humans show interregional differences in cortical (S-Ctx mouse, T-Ctx human) and hippocampal (dorsal mouse, anterior human) glycans (see Supplementary Tables 1–2 and Supplementary Figure 1). These regional CS-GAG differences drive both **(D, G)** hypersulfation of hippocampal CS-GAGs, and **(E, H)** reduced hippocampal A:C ratio of ECM maturation. Data shown as mean ± standard deviation (SD). Abbreviations: hCS, human chondroitin sulfates; mCS, human chondroitin sulfates; CS-GAGs, chondroitin sulfate-glycosaminoglycans; hECM, human extracellular matrix; mECM, mouse extracellular matrix; Hip, hippocampus; LC-MS/MS, liquid chromatography-tandem mass spectrometry; PNN, perineuronal net; T-Ctx, occipitotemporal gyrus of the temporal cortex; S-Ctx, somatosensory cortex; WFA, *Wisteria floribunda* agglutinin.

Perineuronal nets (PNNs) are extracellular matrix (ECM) structures that condense around the soma and proximal dendrites of key populations of neurons in the brain, including those involved in cognitive and memory-forming circuits in the cortex and hippocampus (Figure 1A; Fawcett et al., 2022). These specialized, lattice-like matrices are comprised of chondroitin sulfate proteoglycans (CSPGs) and their CS-glycosaminoglycan (GAG) sugar attachments. The heavily sulfated PNN CS-GAGs have been shown to both stabilize neuronal synapses and act as physicochemical diffusion barriers surrounding the neuronal soma (Christensen et al., 2021; Hanssen and Malthe-Sørenssen, 2022). The introduction of sulfate groups to the CS unit, including non-sulfated CS-O (0S), mono-sulfated CS-A (4S) and CS-C (6S), and di-sulfated CS-D (2S6S) and CS-E (4S6S) (Figure 1B) isomers, produces variably charged CS modules along the CS-GAG chain (Fawcett and Kwok, 2022).

Importantly, the relative abundance of each non-, mono-, and di-sulfated CS isomer incorporated into PNN CS-GAGs and other CS-GAG rich matrices in the brain directly impact the biological activity of the underlying circuitry. Specifically, mono-sulfated CS-A (4S) and CS-C (6S) isomers influence neuronal plasticity, firing patterning, and mechanical tissue rigidity (Miyata et al., 2012; Ryu et al., 2021). In contrast, the non-sulfated CS-O (0S) isomer plays a role in regulating the diffusion of charged molecules throughout the brain parenchyma and cation availability to the neuronal surface (Syková and Nicholson, 2008; Hrabetová et al., 2009). Finally, the di-sulfated CS-D (2S6S) and CS-E (4S6S) isomers offer selective binding sites for various extracellular proteins (e.g., growth factors, guidance cues, cytokines) involved in circuit maturation and maintenance (Djerbal et al., 2017). Although studies clearly support a role for PNNs and their associated CS-GAGs in modulating the formation and maintenance of neurocircuits in rodents (Sorg et al., 2016; Testa et al., 2019; Carulli and Verhaagen, 2021), it is logical to ask whether the results from these pre-clinical studies are recapitulated in (and thus translate to) higher order species.

The overarching goal of this perspective article is to explore the translatability of rodent PNN CS-GAG studies to that of healthy and diseased humans. We first highlight the interregional differences in brain CS-GAGs originally reported in mice (Scarlett et al., 2022), and then test whether these core establishments translate to human brain anatomy. We then review in detail more recent findings that brain CS-GAGs are altered in postmortem tissue from donors with dementia, and then further investigate whether these findings persist across the neuroanatomical distribution of AD neuropathology throughout the demented brain, irrespective of interregional differences in baseline CS-GAG composition. By evaluating the translational value of pre-clinical experimental models to accurately reproduce AD-associated CS-GAG changes, we provide thoughtful guidance to methodologies capable of recapitulating these matrix reorganization events. Recognizing the limitations of rodent models to study human-driven neurological diseases increases the likelihood of developing translational treatments for the prevention and/or reversal of AD.

## 2 Testing the translational relevance of interregional CS-GAG variations across species

Although *Wisteria floribunda* agglutinin (WFA) lectin is commonly considered a universal PNN label, recent evidence now illuminates previously unacknowledged biases on its detection of PNN CS-GAGs (Härtig et al., 2022). Specifically, WFA preferentially recognizes and binds to the non-sulfated CS-O (0S) isomer (Nadanaka et al., 2020), suggesting that PNN/CS-GAG labeling across brain regions (e.g., cortex and hippocampus) may be dependent on region-specific differences in baseline CS-GAG sulfation patterning. Indeed, CS glycan analysis from mouse dorsal hippocampus (Hip) and somatosensory cortex (S-Ctx; Figure 1A) show significant variations in baseline CS-GAG sulfation patterns between these two brain regions (Figure 1C; *p* < 0.0001; see Scarlett et al., 2022; Supplementary Table 1). Mouse S-Ctx exhibits a 2.4x (*p* < 0.0001) higher abundance of the WFA-targeted non-sulfated CS-O (0S) isomer compared to the adjacent Hip, matching the distribution of WFA-labeled PNNs (Figure 1A). However, the mouse Hip displays a 2.2x (*p* < 0.0001) increase in the neuroplastic CS-C (6S) isomer compared to the adjacent S-Ctx. The overall reduction in non-sulfated CS-O (0S) and corresponding increase in sulfated CS isomers drives CS-GAG hypersulfation in the hippocampus (Figure 1D; *p* < 0.0001, see Scarlett et al., 2022). Additionally, the increase in neuroplastic CS-C (6S) isomer (Miyata et al., 2012; Moeendarbary et al., 2017), and corresponding decrease in mature CS-A (4S) isomer (Miyata et al., 2012), resulted in a decrease in the A:C ‘ECM maturation’ ratio in the hippocampus (Figure 1E; *p* < 0.0001; see Scarlett et al., 2022). These findings suggest that the hippocampus may exhibit elevated and region-dependent PNN/CS-GAG hypersulfation, a juvenile matrix environment, and enhanced neuronal plasticity compared to the cortex, thus inspiring us to now ask the following questions:

1. *Are interregional CS-GAG sulfation patterns a response to the specific environmental needs of the enmeshed neuronal circuitry*?
2. *How translatable are mouse interregional CS-GAG brain patterns to higher-order primates*?

Before beginning to assess the functional significance of these interregional CS-GAG distinctions, we must first evaluate the translatability of these pre-clinical findings to human brain anatomy. Using LC–MS/MS (Supplementary Methods; Supplementary Figure 1; Alonge et al., 2019), we determined the relative abundance of each non-, mono-, and di-sulfated CS isomer extracted from the anterior hippocampus (Hip) and adjacent occipitotemporal gyrus of the temporal cortex (T-Ctx) in a cohort of postmortem human brain tissue (Supplementary Table 2). We report here the novel discovery that humans recapitulate many of the interregional CS-GAG sulfation differences as first reported in mice (Figure 1C–E; Scarlett et al., 2022). These regional differences included both a 2.1x (*p* < 0.0001) increase in T-Ctx non-sulfated CS-O (0S) and a 1.4x (*p* < 0.0001) increase in Hip mono-sulfated CS-C (6S) isomer (Figure 1F; Supplementary Table 1). Similar to mice, the overall reduction in the non-sulfated CS isomer and corresponding increases in sulfated CS isomers drove both PNN/CS-GAG hypersulfation (Figure 1G; *p* = 0.0006) and reduced matrix maturation (Figure 1H; *p* < 0.0001) in human hippocampus. A comprehensive exploration of the roles that each individual isomer plays in distinguishing human brain regions was evaluated using averaged representational dissimilarity matrices balanced across classes with subsampling (Figure 1I; Supplementary Methods; Kriegeskorte et al., 2008; Tovar et al., 2020). From this analysis, we determined that interregional differences in human brain CS-GAGs were mostly influenced by CS-C (6S) (63 ± 11% of the optimized model), CS-O (0S) (27 ± 15% of the optimized model), with minimal influence by CS-A (4S) (10 ± 11% of the optimized model; Figure 1I). These results confirm that the top 2 isomers driving interregional PNN/CS-GAG differences between cortex and hippocampus were both the non-sulfated CS-O (0S) and the mono-sulfated CS-C (6S) isomers (Figure 1I; *p* < 0.0001).

**Figure 2:**
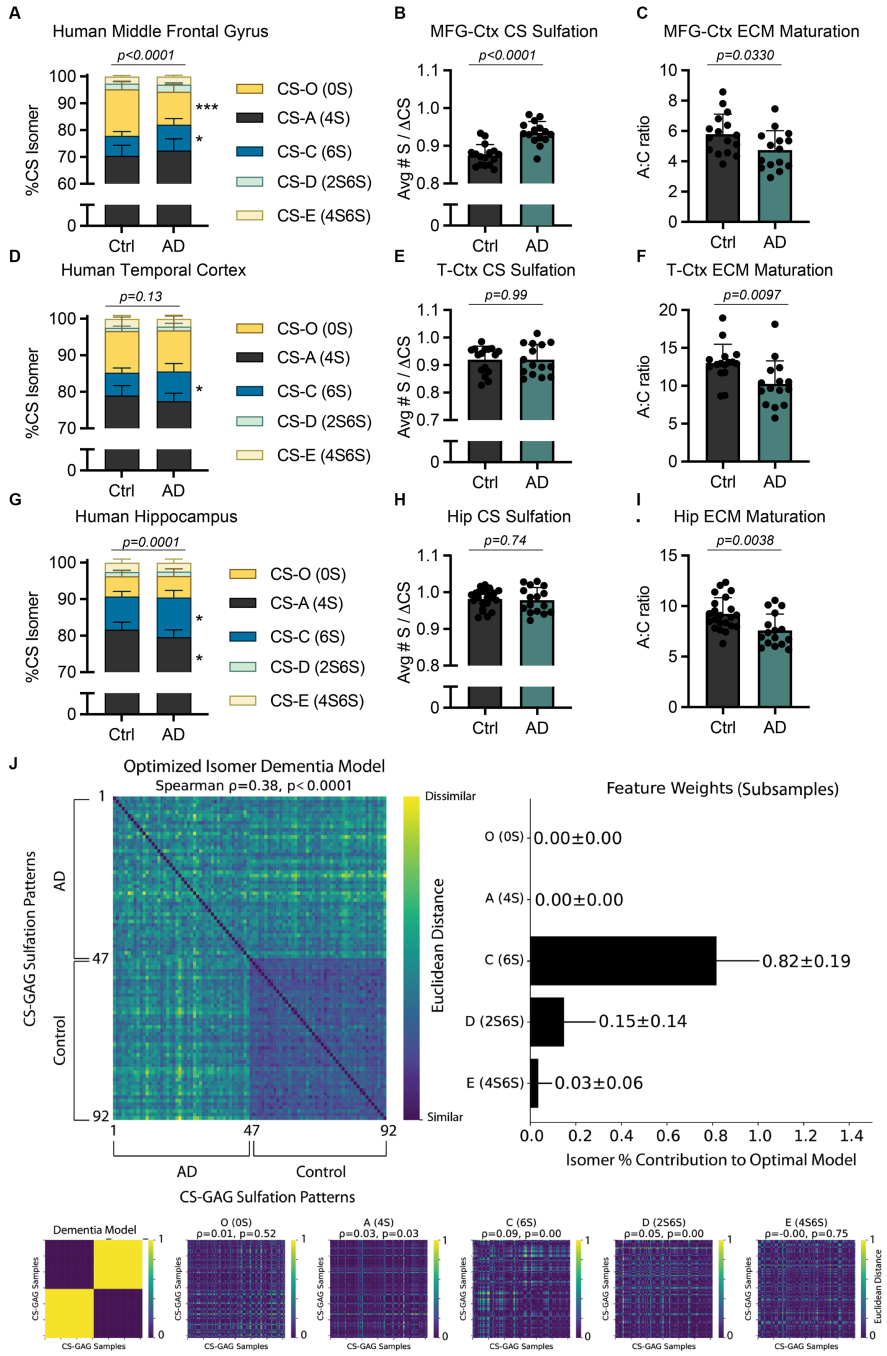
AD-associated interregional PNN/CS-GAG changes in human postmortem brain tissue. CS-GAGs were isolated and analyzed by LC-MS/MS from the **(A-C)** middle frontal gyrus (see Logsdon et al., 2022), **(D-F)** occipitotemporal gyrus of the temporal cortex, and **(G-I)** anterior hippocampus in demented and non-demented control subjects (see Supplementary Tables 2–3) and Supplementary Figure 1). **(B,E,H)** CS-GAG hypersulfation and **(C, F, I)** FIGURE 2 (Continued)the A:C ratio of ECM maturation were also assessed. Data shown as mean ± standard deviation (SD). Abbreviations: AD, Alzheimer’s disease; CS-GAGs, chondroitin sulfate-glycosaminoglycans; Ctrl, controls; Hip, anterior hippocampus; LC-MS/MS, liquid chromatography-tandem mass spectrometry; PNN, perineuronal net; MFG-Ctx, middle frontal gyrus; T-Ctx, occipitotemporal gyrus of the temporal cortex.

Our finding that rodents and humans exhibit hypersulfated, negatively charged extracellular environments selectively within the hippocampus may indeed represent a biological response to specific neurocircuitry and neuronal activity within that brain region. Notably, PNN CS-GAGs have been shown to enmesh fast-spiking neurons and aid in rapid local buffering of excess cation exchanges (Härtig et al., 1999). As such, hypersulfation of hippocampal CS-GAGs may assist with maintaining elevated firing activity unique to this brain region. Although the neocortex is believed to be involved in memory retrieval and systems consolidation (Haist et al., 2001; Preston and Eichenbaum, 2013), functional magnetic resonance imaging (fMRI) in humans demonstrates the middle frontal gyrus and medial temporal cortex exhibit variable and temporally graded activity changes during memory recall (Smith and Squire, 2009; Tallman et al., 2022). In contrast, the hippocampus displays a consistent elevation of neural activity during both the initial memory formation and memory retrieval over time (Tallman et al., 2022). This constant state of activity is supplemented by pre-onset spiking prior to memory establishment (Urgolites et al., 2020) and sequence replay during sleep for memory retention (Schapiro et al., 2018). These results show that continuous hippocampal activity is required for memory formation and retrieval, regardless of time, and supports the multiple-trace theory of episodic memory formation. We now predict that negatively charged hippocampal CS-GAGs in rodents (Figure 2C) and humans (Figure 2F) may play a critical role in supporting these biological processes. Furthermore, disruptions to this hypersulfated matrix environment may be linked to aberrant neuronal activity underlying neurological disorders.

## 3 Determining the effects of AD on CS-GAG patterning in postmortem human brain tissue

The influence of AD on PNN/CS-GAGs in the demented brain was reported over 35 years ago; a study by Kobayashi et al. in 1989 made a seminal observation that postmortem brain tissue from demented subjects exhibited reduced *Vicia villosa* agglutinin (VVA) lectin labeling of PNN CS-GAGs (Kobayashi et al., 1989). A second translational study led by Baig et al. in 2005 further supported this finding by reporting a 70% reduction in PNN CS-GAGs in postmortem AD brain tissues when labeled using WFA lectin (Baig et al., 2005). However, it was only within the last 5 years that quantitative mass spectrometry has interrogated whether PNN CS-GAG sulfation patterns themselves are altered in this neurodegenerative disease.

Specifically, our lab reported a significant shift in cortical CS-GAG sulfation patterns isolated from postmortem middle frontal gyrus (MFG-Ctx) in demented subjects when compared to non-demented controls (Figure 2A; *p* < 0.0001; see Logsdon et al., 2022; Supplementary Table 3). Our report showed the MFG-Ctx from AD brain donors exhibited a 29.5% (*p* = 0.0002) decrease in the non-sulfated CS-O (0S) isomer, and a corresponding 1.3x (*p* = 0.02) increase in the mono-sulfated CS-C (6S) isomer, compared to non-demented controls. These shifts resulted in both hypersulfation of MFG-Ctx CS-GAGs (Figure 2B; *p* < 0.0001; see Logsdon et al., 2022) and a significant decrease in the A:C ratio of ECM maturation (Figure 2C; *p* = 0.0330; see Logsdon et al., 2022). Considering that the CS-C (6S) isomer is also involved in PNN destabilization and increased mechanical pliability of brain tissue (Miyata et al., 2012; Miyata and Kitagawa, 2016; Ryu et al., 2021), the results from this initial study characterized the AD brain as having a more developmental CS-GAG matrix supportive of neurocircuit plasticity and reorganization.

The paradoxical shift to favor developmental CS-GAG matrices in the demented brain appeared to occur exclusively in brain regions reflective of AD neuropathology accumulation, since CS-GAGs isolated from the adjacent cerebellar cortex did not exhibit changes in CS-GAG sulfation patterns (Logsdon et al., 2022). However, the cerebellar cortex also displays a surprising 1.8x increase in CS-C (6S) compared to the MFG-Ctx, which now raises additional mechanistic questions that investigate potential linkages between interregional differences in baseline CS-GAG patterning and the progression of AD:

1. *Are brain regions that exhibit elevated baseline of CS-C (6S) ‘protected’ against CS-GAG changes in AD?*
2. *Or alternatively, does the AD-associated increase of CS-C (6S) occur independently to differences in baseline CS-GAG patterning?*

Based on the observation that healthy control subjects exhibit differences in baseline CS-GAG sulfation patterns between the hippocampus and cortex (Figure 1F), including a 1.4x greater expression of CS-C (6S) in anterior Hip when compared to the T-Ctx (Figure 1F), we next determined whether AD influences CS-GAG patterning similarly between these two brain regions, irrespective to interregional baseline CS-GAG differences. Using LC–MS/MS to isolate and quantify the relative abundance of CS isomers isolated from T-Ctx and Hip of postmortem brain tissue (Supplementary Figure 1; Table 2), we report that AD associates with a significant increase in CS-C (6S) isomer in both of these brain regions (Supplementary Table 3), including a 1.3x (*p* < 0.05) increase in the T-Ctx and 1.2x (*p* = 0.02) increase in the anterior Hip (Figures 2D,G). Although AD-associated increases in CS-C (6S) resulted in a significant decrease in the A:C ratio of ECM maturation in both brain regions (Figures 2F,I), this effect was not linked to AD-associated changes in CS-O (0S) or hypersulfation of CS-GAGs (Figures 2E,H) that were originally reported for the MFG-Ctx (Figure 2B). Multivariate analyses of MFG-Ctx, T-Ctx, and Hip using averaged subsampled dissimilarity matrices coupled with Bayesian optimization also revealed that CS-C (6S), but not CS-O (0S), was uniquely related to clinical diagnoses of dementia in patients (82 ± 19% of the optimized model; Figure 2J).

Mechanistically, the increase in mono-sulfated CS-C (6S) observed between all three AD affected brain regions, regardless of interregional baseline CS-GAG differences, is of particular importance when understanding the role of CS-GAGs in driving this disease. In mice, the CS-C (6S) isomer is highest before the critical period in neurodevelopment (25.5–27.5% at P0) and supports neurocircuit plasticity during this time (Miyata et al., 2012; Alonge et al., 2019). After closure of the critical period, the expression of CS-C (6S) significantly drops (2.3–2.9%) after 3 months of age (Miyata et al., 2012; Alonge et al., 2019), which allows for PNN maturation and resulting circuit stability. In contrast to these age-related changes reported in mice, AD in humans associates with a paradoxical *increase* in the developmental CS-C (6S) isomer (Supplementary Table 3; Logsdon et al., 2022). We predict that the increase in CS-C (6S) may drive a spike in both neuroplasticity and reorganization within these brain regions, as observed with the consistent decrease in the A:C ratio of ECM maturation (Figures 2C,F,I). Moreover, this effect may partially contribute to circuit dysfunction and reorganization as well as neurodegenerative-associated neuropathology and neurotoxicity.

Intriguingly, we noticed that the di-sulfated CS-D (2S6S) isomer also appeared to have some influence in the dementia diagnosis within our patient population (15 ± 14% of the optimized model; Figure 2J). The CS-D (2S6S) isomer was recently recognized as the major hippocampal isomer increased in the PS19 mouse model of tauopathy (Logsdon et al., 2024) and may provide valuable insight into how the brain biologically defends against neurodegeneration (please see Discussion below). Collectively, these findings implicate both CS-C (6S) and CS-D (2S6S) as the major isomers increased in all three brain regions associated with AD neuropathology accumulation in postmortem brain tissue (i.e., MFG-Ctx, T-Ctx, and Hip), and that this effect appears independent to interregional baseline differences in CS-GAG patterning.

## 4 Discussion

By first determining whether humans exhibit comparable interregional differences in brain CS-GAG sulfation patterning as mice, this perspective article takes a necessary step in establishing translational relevance in brain CS-GAG changes between rodents and humans. We first show that both species exhibit a significant decrease in hippocampal non-sulfated CS-O (0S) isomer, and corresponding increase in mono-sulfated CS-C (6S) isomer, when compared to the adjacent cortex (Figure 1), and that this reduction establishes a strong and noteworthy matrix charge difference between the two regions. The biological relevance of these interregional CS-GAG differences is of intense interest and value to the biomedical community with respect to baseline cytoarchitecture and connectivity, especially considering the regional influence of extracellular matrices on neuronal functioning. PNN CS-GAGs have been shown to enmesh fast-spiking neurons and the CS-GAG sulfate attachments aid in the rapid buffering of extracellular cations (Härtig et al., 1999). Moreover, changes in PNN composition has been shown to destabilize the PNN matrices themselves and induce aberrant firing of the underlying neurons (Miyata et al., 2012). As such, the unique composition and hypersulfation of CS-GAGs specific to the hippocampus may assist with maintaining neuronal firing activity and circuit plasticity selective to this brain region.

Not only can PNNs and other extracellular matrices influence firing properties of the underlying neurons, but the neurons themselves can dictate reorganization of the surrounding extracellular environment in response to activity needs. Work by Devienne et al. (2021) provides compelling evidence that parvalbumin (PV) neurons are direct mediators of PNN CS-GAG biosynthesis; whereas activation of PV neurons increases PNN CS-GAG formation, inhibition of this same neuronal population results in matrix repression (Devienne et al., 2021). We hypothesize that the loss of VVA and WFA-labeled PNN CS-GAGs in demented brain tissue (Kobayashi et al., 1989; Baig et al., 2005) may be driven by hypoactivity of the underlying neurocircuitry. Although the hippocampus is hyperactive in prodromal AD, hypoactivity supersedes as the disease progresses (Targa Dias Anastacio et al., 2022). Here, we show that brain tissue from donors with AD dementia exhibit a significant increase in mono-sulfated CS-C (6S) isomer within the MFG-Ctx, T-Ctx, and hippocampus compared to non-demented controls (Figure 2; Logsdon et al., 2022), but only the MFG-Ctx exhibits a perplexing decrease in the non-sulfated CS-O (0S) isomer and hypersulfation of CS-GAGs. These region-dependent differences may be driven by the spatiotemporal progression of AD and resulting neurodegeneration and hypoactivity of the afflicted neurons. Since the MFG-Ctx accumulates pTau in the later stages of AD neuropathologic progression (Braak and Braak, 1991), this region may uniquely exhibit (1) retained basal activity as the disease progresses, and (2) undergo reduced deactivation by the connecting hypoactive hippocampal circuits (Celone et al., 2006), which are targeted early in the disease. While our understanding of how neuronal activity influences PNN CS-GAG sulfation patterning is currently unknown, hyperactive neurons in the MFG-Ctx may increase sulfation of the surrounding CS-GAGs as a biological defense response to increased neuronal activity.

Since CS-GAGs comprise both interstitial and PNN matrices, it is difficult to determine the specific roles of each matrix subtype in supporting the needs of the enmeshed firing neurons. Indeed, both stable PNN/CS-GAG matrices and diffuse CS-GAG-containing interstitial pericellular coats may exhibit overlapping functions in regulating the enmeshed neuron. PNNs themselves exist as a spectrum of both diffuse and structured morphologies throughout rodent and human brain (Alonge et al., 2020; Scarlett et al., 2022), the most prominent example showcasing this range is observed in hippocampal CA1 (structured PNNs, open arrow) vs. CA2 (unstructured PNNs, closed arrow) subregions (Figure 1A). As such, the mass spectrometry analyses utilized here assess the relative abundance of CS isomers isolated from all matrix subtypes. Although results from our LC–MS/MS methodology match the CS-GAG sulfation patterning reported using PNN/CS-GAG fractionation (Alonge et al., 2019), suggesting that CS-GAG signatures derived by LC–MS/MS are driven predominantly by PNNs, it still provides the average sulfation patterning from all matrix subtypes contained within the tissue region. By sampling structured and unstructured PNNs, in addition to interstitial pericellular coats, our method takes into account the influence of multiple matrix subtypes interacting with the underlying circuitry and represents a more inclusive approach to analyzing CS-GAG-containing matrices within the brain.

In humans, shifts in CS-GAG sulfation patterns (Figure 2; Logsdon et al., 2022) and PNN CS-GAG abundance (Brückner et al., 1999; Morawski et al., 2010, 2012) closely follow pTau accumulation and AD progression, but this core finding has only been partially translated to a mouse model of tauopathy. We recently tested whether PS19 and/or Tau4RTg2652 mice exhibit PNN changes in association with pTau burden over time. Whereas Tau4RTg2652 mice did not show changes in stable PNNs in the presence of high pTau (soluble), we did observe strong correlations between accumulation of pTau (insoluble), gliosis, and PNN CS-GAG loss in aged PS19 mice (Logsdon et al., 2024). However, the loss of hippocampal PNN CS-GAGs in aged PS19 mice did not correlate with an increase in CS-C (6S) isomer expression as observed in humans (Figure 2), and thus we conclude that increased expression of CS-C (6S) is not required for PNN deglycosylation or pTau expression in this mouse line. Instead, we reported a strong correlation between PNN deglycosylation, gliosis, and neurodegeneration with an increase in di-sulfated CS-D (2S6S) isomer (Logsdon et al., 2024). The CS-D (2S6S) isomer is a unique CS variant associated with neuroregenerative properties, including increasing neurite bearing neurons (Clement et al., 1999), their process length (Clement et al., 1999), and migration in hippocampal cell cultures (Shida et al., 2019). We now predict that the increase in CS-D (2S6S) isomer in our demented population (Figure 2) and in the PS19 mouse model of tauopathy (Logsdon et al., 2024) may represent a biological defense to AD-associated neurodegeneration in both humans and mice.

Although mice do not appear to exhibit an endogenous increase in the CS-C (6S) isomer with aging (Foscarin et al., 2017; Yang et al., 2021), or an increase in response to pTau expression (Logsdon et al., 2024), they still may be utilized as critical translational tools to studying matrix-related mechanisms underlying AD pathophysiology and neurotoxicity in appropriate conditions. Researchers should consider leveraging the low abundance of CS-C (6S) expression in mice by employing biochemical methods to increase expression of CS-C (6S) within the mouse system. Miyata et al. (2012) showed that transgenic mice overexpressing the 6S-sulfotransferase (C6ST-1) resulted in a 4.2x-increase in CS-C (6S) isomer expression in adult P60 mice, an effect that is linked to both destabilization of WFA^+^ PNN matrices and aberrant firing of the underlying circuitry (Miyata et al., 2012). A second method to increase localized expression of CS-C (6S), Yang et al. (2021) employed AAV viral overexpression to artificially increase expression of CS-C (6S) within the adult mouse brain (Yang et al., 2021). Both transgenic mice and AAV-mediated overexpression of the CS-C (6S) isomer may hold great value in determining the role this specific isomer plays in underlying the pathogenesis of AD. In conclusion, this perspective article provides novel insights into the translatability of utilizing murine models to represent both healthy and diseased human brain, the results of which are critical in establishing a relevant foundation for future AD therapy development.

## Data availability statement

The original contributions presented in the study are included in the article/Supplementary material, further inquiries can be directed to the corresponding authors.

## Ethics statement

Ethical approval was not required for the studies involving humans because the use of de-identified, postmortem human brain samples do not meet the federal definition of human subject research and do not fit the criteria involving human subjects. The studies were conducted in accordance with the local legislation and institutional requirements. The human samples used in this study were acquired from UW BioRepository and Integrated Neuropathology (BRaIN) laboratory and the Kaiser Permanente Washington Adult Changes in Thought (ACT) study. Written informed consent to participate in this study was not required from the participants or the participants’ legal guardians/next of kin in accordance with the national legislation and the institutional requirements. The animal study was approved by UW’s Institutional Animal Care and Use Committee. The study was conducted in accordance with the local legislation and institutional requirements.

## Author contributions

AH: Conceptualization, Data curation, Investigation, Methodology, Supervision, Writing – original draft, Writing – review & editing. KF: Data curation, Funding acquisition, Investigation, Methodology, Resources, Supervision, Writing – review & editing. AK: Conceptualization, Investigation, Supervision, Writing – original draft, Writing – review & editing. JL: Conceptualization, Investigation, Supervision, Writing – original draft, Writing – review & editing. JS: Conceptualization, Data curation, Funding acquisition, Investigation, Methodology, Project administration, Resources, Supervision, Writing – review & editing. CK: Data curation, Funding acquisition, Investigation, Project administration, Resources, Supervision, Validation, Writing – review & editing. DT: Conceptualization, Data curation, Formal analysis, Funding acquisition, Investigation, Methodology, Project administration, Resources, Software, Supervision, Validation, Visualization, Writing – original draft, Writing – review & editing. KA: Conceptualization, Data curation, Formal analysis, Funding acquisition, Investigation, Methodology, Project administration, Resources, Software, Supervision, Validation, Visualization, Writing – original draft, Writing – review & editing.
