# Supplementary material for: Assessing translational applicability of perineuronal net dysfunction in Alzheimer’s disease across species
Source: Front Neurosci. 2024 Apr 30;18:1396101. doi: 10.3389/fnins.2024.1396101 (PMC11091253; doi:10.3389/fnins.2024.1396101)
Supplement: Supplementary file 1 [file Presentation_1.pdf]

Supplementary Material

Assessing translational applicability of perineuronal net dysfunction across species in Alzheimer's disease

Aarun S. Hendrickson[1,2], Kendra L. Francis[2,3], Asmit Kumar[1,2], Jaden P. Le[1,2], Jarrad M. Scarlett[2,3], C. Dirk Keene[4], David A. Tovar[5#*], Kimberly M. Alonge[1,2#*]

[1]Medicinal Chemistry, University of Washington, Seattle, WA, 98195, USA.
[2]Department of Medicine, University of Washington Medicine Diabetes Institute, Seattle, WA, 98109, USA.
[3]Department of Pediatric Gastroenterology and Hepatology, Seattle Children's Hospital, Seattle, WA, 98145, USA.
[4]Department of Laboratory Medicine and Pathology, University of Washington, Seattle, WA, 98195, USA.
[5]Department of Psychology, Vanderbilt University, Nashville, TN, 37325, USA.
[#] **equal senior author contribution**

*** Correspondences:**
Kimberly M. Alonge, PhD
kalonge@uw.edu

David A. Tovar, MD, PhD
david.tovar@vanderbilt.edu

1. Supplementary Methods

1.1. Animals
1.2. Mouse brain collections
1.3. Mouse brain immunofluorescence
1.4. Human brain CS-GAG digestion and CS disaccharide isolation
1.5. Mass spectrometry quantification of the relative abundance of CS isomers
1.6. Dissimilarity matrix comparisons
1.7. Statistics

2. Supplementary Figures

2.1. *Supplementary Figure 1: LC-MS/MS of human brain tissue*

3. Supplementary Tables

3.1. *Supplementary Table 1. Mouse vs human interregional CS-GAG sulfation patterning*
3.2. *Supplementary Table 2. Human patient characteristics*
3.3. *Supplementary Table 3. Effects of AD on Human CS-GAGs*

1 Supplementary Methods

1.1. Animals

A wild-type mouse (P42, male) from our in-house C57Bl/6 background was used for the representative WFA labeling of mouse brain PNNs. The mouse had *ad libitum* access to chow and water and resided in temperature-controlled rooms under 12 h-dark: 12 h-light cycles. All procedures were performed in accordance with NIH guidelines for the care and use of laboratory animals and were approved by the Institutional Animal Care and Use Committee (IACUC) at the University of Washington (Seattle, Washington), and all experiments were conducted in accordance with the National Institutes of Health Guide for the Care and Use of Laboratory Animals. All experiments reported are in compliance with ARRIVE guidelines.

1.2. Mouse brain collections

The mouse was anesthetized with ketamine and xylazine and cardiac perfused with 0.1 M phosphate buffered saline (PBS) followed by sequential perfusion using 4% PFA in 0.1 M PBS. The brain was extracted, post-fixed in 4% paraformaldehyde (PFA) for 48 h at 4°C, cryopreserved in 30% sucrose in 0.1 M PBS, and frozen in optimal cutting temperature (OCT) solution on dry ice. The brain was stored at -80°C and equilibrated at -20°C prior to sectioning. The brain was cut on a Leica CM1950 cryostat at 30 μm-thick serial sections and stored in 0.1 M PBS supplemented with 0.02% sodium azide at 4°C as free-floating sections.

1.3. Mouse brain immunofluorescence

30 μm-thick mouse coronal sections underwent antigen retrieval in 10 mM trisodium citrate (pH 8.5) for 20 min at 90°C. Immunostaining of the free-floating tissue was performed by permeabilizing the free-floating tissues in 0.1 M PBS + 0.2% Triton X-100 for 30 m, followed by tissue blocking in 0.1 M PBS + 0.05% Triton X-100 (PBS-T) + 10% normal donkey serum (Jackson ImmunoResearch) for 2 h at 37°C. Sections incubated overnight at 4°C using 1:1,000 dilution of biotin labeled *Wisteria floribunda* agglutinin (WFA) (Sigma: L1516) in PBS-T + 1% donkey serum. The next day, sections were washed and incubated in a 1:1,000 dilution of Alexa-fluor-conjugated secondary antibody in PBS-T + 1% donkey serum for 2 h. Sections were then counterstained for DAPI, mounted, and cover slipped using Fluoromount-G (ThermoFisher, 4958-02). Tile imaging was conducted with a Keyence BZ-800 inverted microscope using a 10X objective.

1.4. Human brain CS-GAG digestion and CS disaccharide isolation

Human brain sections containing both anterior hippocampus and adjacent occipitotemporal gyrus of the temporal cortex were provided as 4 μm-thick formalin-fixed paraffin embedded (FFPE) sections that were mounted onto slides. FFPE tissues were de-waxed using xylenes (Thermo Fischer Scientific, 1330-20-07, Waltham, MA) and a series of 100%, 95%, 70%, and 50% graded ethanol washes. Slides were then washed 1x in 0.1 M PBS, 3x in Optima LC/MS-grade water (Thermo Fisher Scientific, 7732-18-5, Waltham, MA), and 1x in 50 mM ammonium bicarbonate (pH 7.6; Sigma, 09830, Burlington, MA). ChondroitinaseABC (ChABC) enzyme (Sigma, C3667, Burlington, MA) was reconstituted at a concentration of 0.5 U/mL in 50 mM ammonium bicarbonate (pH 7.6). Regional isolations were performed on the tissue sections by scratching non hippocampal and non-occipitotemporal gyrus of the temporal cortex tissue from the slides, followed by hydrophobic pen isolations directly on the slide separating the two regions. The reconstituted ChABC enzyme was then added to each region on the slide (~100-200 μL per region) and the slides were then incubated at 37°C for 24 h. Afterward, the

digested glycans were collected from the slide and spun at 14,000xg for 10 m to remove any tissue debris. The supernatant was collected and dehydrated using a Thermo Fisher Scientific SpeedVac Concentrator, and the resulting product was reconstituted in 30 µL of LC/MS-grade water for mass spectrometry analysis.

1.5. Mass spectrometry quantification of the relative abundance of CS isomers

Isolated CS isomers were analyzed using a triple quadrupole mass spectrometer equipped with an electrospray ion source (Waters Xevo TQ-S) operated in negative mode ionization. LC-MS/MS was performed using a Waters Acquity I-class ultra-performance liquid chromatographic system (UPLC) coupled to the same Waters Xevo TQ-S system. Disaccharides were resolved by porous graphitic chromatography (Hypercarb column; 2.1 x 50 mm, 3 µm; ThermoFisher) and assigned multiple reaction monitoring (MRM) channels: CS-A (4S), *m/z* 458 > 300; CS-C (6S), *m/z* 458 > 282; CS-E (4S6S), *m/z* 538 > 300; CS-D (2S6S), *m/z* 268 > 282; CS-O (0S), *m/z* 378 > 175. MassLynx software version 4.1 (Waters) was used to acquire and quantify all data. Under the conditions described above, the ratios between peak areas produced from equimolar CS standard runs were normalized to the highest peak intensity and relative quantification of each CS isomer within a sample was achieved using a modified peak area normalization function (Alonge et al., 2019). Each CS isomer was expressed as a relative abundance percentage of the total CS isomer composition within each sample. Acetonitrile (optima LC/MS-grade) and all other reagents were obtained from Fisher Scientific.

1.6. Dissimilarity matrix comparisons

Representational similarity analysis (Kriegeskorte et al., 2008) was used to investigate the dissimilarity in CS isomer sulfation patterns between brain regions of healthy controls, as well as within brain regions of healthy controls and individuals with dementia. Initially, we constructed model dissimilarity matrices for the hippocampus (assigned as 0) and cortex samples (assigned as 1) within healthy controls, modeling uniformity within each brain region. A similar approach was applied to samples from individuals with dementia and their age-matched controls, with dementia patients and controls assigned values of 1 and 0, respectively. This modeling was aimed at identifying unique CS isomers that significantly differentiate between the samples based on the specified conditions, rather than explaining the total variance. To ensure a balanced comparison between cortical and hippocampal samples and between dementia patients and controls, we implemented a subsampling procedure. This involved randomly selecting samples from the group with more samples to match that of the group with less samples as to equalize group sizes, repeated 20 times to mitigate subsample bias. Our reported results represent the averages across these subsamples. To accurately capture differences in CS isomer sulfation patterns related to brain region and dementia status, beyond mere abundance, we normalized each isomer through min/max normalization, assigning 0 to the least abundant and 1 to the most abundant isomers. We then generated dissimilarity matrices for each isomer using Euclidean distance and calculated the Spearman correlation between these isomer-specific matrices and their respective model matrix. Using the Spearman rho as initial values, we employed Bayesian optimization to refine the linear combination of isomers, optimizing their correlation with the model matrix (representing either brain region or dementia status). We assigned a weight of 0 to negative correlations, while positive rho values were normalized to sum to 1, ensuring the combined weights of all isomers contributed evenly. This optimization was performed over 50 steps for each of the 20 subsamples, with 100 random initializations per subsample to account for the stochastic nature of the process. The optimal weights from these runs were averaged, alongside their standard deviations. The mean optimized isomer matrix was then correlated with the model matrix using Spearman correlation, the results of

which are presented in **Figure 1I** and **Figure 2J**. This methodology allowed us to systematically identify and quantify the isomer patterns most relevant to distinguishing between brain regions and dementia status in our samples.

1.7. Statistics

A two-way ANOVA with matched isomers and paired brain regions (for interregional comparisons within species) and a two-way ANOVA with matched isomers only (for effects of AD) fit full interaction term models with multiple comparisons corrected using Šidák test in GraphPad Prism® 8.0 (Graph Pad Software, Inc., La Jolla, CA). Comparison of CS-GAG sulfation and A:C ratio of ECM maturation used either paired t-test (for interregional comparisons within species) or non-paired t-test (for effects of AD). Error bars represent the standard deviation (SD) of the mean.

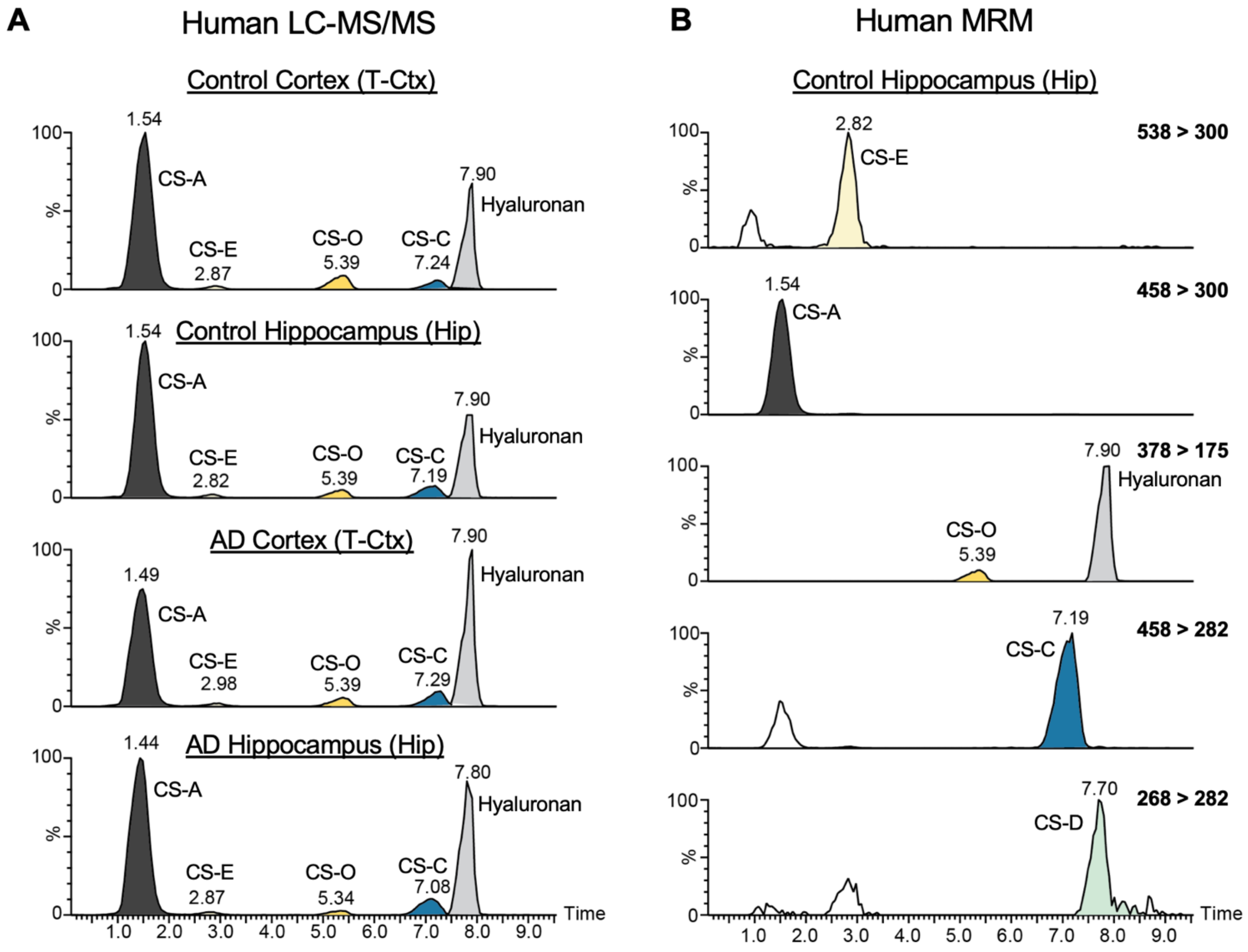

Supplemental Figure 1. LC-MS/MS and MRM of human brain tissue. Representative images of (**A**) compiled LC and (**B**) individual MRM chromatograms of CS isomers by mass spectrometry. CS-GAGs were digested from postmortem FFPE tissues and representative chromatograms from (**A**, *top 2 panels*) control cortex and hippocampus of the same female donor, and (**A**, *bottom 2 panels*) demented cortex and hippocampus of the same female donor, show increased CS-C (6S) (*blue*) in the hippocampus and increased CS-O (0S) (*yellow*) in the cortex for each patient, respectively. Representative chromatograms from (**A**, *bottom 2 panels*) demented cortex and hippocampus show increased CS-C (6S) (*blue*) when compared to (**A**, *top 2 panels*) control cortex and hippocampus. (**B**) Closer examination of the control hippocampus MRM shows separation of each CS isomer by channel and time. ChABC exhibits Hyaluronidase activity, and the release of hyaluronan disaccharides can be used as a marker for high enzyme activity. Abbreviations: AD, Alzheimer's disease; ChABC, chondroitinaseABC; CS, chondroitin sulfate; CS-GAGs, chondroitin sulfate-glycosaminoglycans; FFPE, formalin fixed paraffin embedded; Hip, anterior hippocampus; T-Ctx, occipitotemporal gyrus of the temporal cortex; LC-MS/MS, liquid chromatography-tandem mass spectrometry; MRM, multiple reaction monitoring.

Supplementary Table 1. Mouse vs human interregional CS-GAG sulfation patterning.

	CS-O (0S)	CS-A (4S)	CS-C (6S)	CS-D (2S6S)	CS-E (4S6S)
**Mouse**					
S-Ctx	16.9 (1.2)	74.9 (1.6)	4.9 (0.3)	2.3 (0.2)	0.9 (0.1)
Hip	7.1 (0.4)	78.1 (0.9)	10.6 (0.5)	3.3 (0.3)	0.9 (0.1)
p-value	$p < 0.0001$	$p < 0.0001$	$p < 0.0001$	$p < 0.0001$	$p = 0.99$
**Human**					
T-Ctx	11.4 (3.8)	79.0 (2.7)	6.3 (1.2)	0.9 (0.5)	2.5 (0.9)
Hip	5.5 (1.6)	81.7 (2.0)	9.1 (1.4)	1.2 (0.5)	2.6 (1.0)
p-value	$p < 0.0001$	$p = 0.0140$	$p < 0.0001$	$p = 0.35$	$p > 0.99$

Mouse interregional CS-GAG sulfation patterning was original published in (Scarlett, et al. 2022) and republished here with copyright permissions. Human T-Ctx and Hip CS-GAG sulfation patterning are of original work for this manuscript. Data is shown as mean (standard deviation). Abbreviations: CS, chondroitin sulfate; CS-GAGs, chondroitin sulfate-glycosaminoglycans; Hip, hippocampus; T-Ctx, occipitotemporal gyrus of the temporal cortex; S-Ctx, somatosensory cortex.

Supplementary Table 2. Brain donor characteristics.

	Non-demented	Demented (AD)
**Demographic Features**		
Number	22	16
Age (years)		
Mean (SD)	84.9 (7.3)	86.1 (7.3)
Median (IQR)	86.0 (78.0-89.8)	87.0 (79.0-91.3)
Sex (Male/Female)	(6/16)	(5/11)
Post-mortem Interval (hours)		
Mean (SD)	6.9 (5.0)	6.9 (4.8)
Median (IQR)	5.3 (3.9-8.6)	5.4 (3.7-8.5)
Matched Hip/T-Ctx regions	16/22	15/16
**AD Neuropathology**		
Number	22/22	16/16
Braak Stage		
B1: Braak 0-II	10	0
B2: Braak III/IV	12	1
B3: Braak V/VI	0	15
Mean (SD)	2.6 (1.0)	5.8 (0.6)
Median (IQR)	3 (2.0-3.3)	6.0 (6.0-6.0)
CERAD Score (neuritic plaques)		
None	10	0
Sparse	5	1
Moderate	7	0
Frequent	0	15
Mean (SD)	0.9 (0.9)	2.9 (0.5)
Median (IQR)	1 (0-2)	3 (3-3)
Age of tissue (years)		
Mean (SD)	18.0 (4.5)	12.9 (3.8)
Median (IQR)	18.0 (16.0-20.3)	14.5 (10.3-16.8)

Supplementary Table 3. Effects of AD-dementia on brain CS-GAGs.

	CS-O (0S)	CS-A (4S)	CS-C (6S)	CS-D (2S6S)	CS-E (4S6S)
**Middle Frontal Gyrus (MFG-Ctx)**					
Controls	17.3 (3.1)	70.4 (3.9)	7.5 (1.6)	2.1 (0.6)	2.6 (0.3)
AD Dementia	12.2 (2.8)	72.4 (4.4)	9.7 (2.3)	2.7 (0.6)	3.0 (0.4)
p-value	$p = 0.0002$	$p = 0.67$	$p = 0.0237$	$p = 0.14$	$p = 0.07$
**Temporal Cortex (T-Ctx)**					
Controls	11.4 (3.8)	79.0 (2.7)	6.3 (1.2)	0.9 (0.5)	2.5 (0.9)
AD Dementia	11.3 (4.0)	77.4 (2.2)	8.1 (2.2)	1.0 (0.9)	2.1 (1.0)
p-value	$p > 0.99$	$p = 0.36$	$p < 0.05$	$p = 0.97$	$p = 0.89$
**Hippocampus (Hip)**					
Controls	5.5 (1.6)	81.7 (2.0)	9.1 (1.4)	1.2 (0.5)	2.6 (1.0)
AD Dementia	5.8 (2.0)	79.6 (2.0)	10.9 (1.9)	1.2 (0.7)	2.5 (1.0)
p-value	$p = 0.99$	$p = 0.0209$	$p = 0.0177$	$p > 0.99$	$p > 0.99$

Human middle frontal gyrus CS-GAG sulfation patterning was original published in (Logsdon, et al. 2022) and republished here with copyright permissions. Human T-Ctx and Hip CS-GAG sulfation patterns are of original work for this manuscript. Data is shown as mean (standard deviation). Abbreviations: AD, Alzheimer's disease; CS, chondroitin sulfate; CS-GAGs, chondroitin sulfate-glycosaminoglycans; Hip, anterior hippocampus; T-Ctx, occipitotemporal gyrus of the temporal cortex; MFG-Ctx, middle frontal gyrus cortex.
